# Disparate Habitual Physical Activity and Dietary Intake Profiles of Elderly Men with Low and Elevated Systemic Inflammation

**DOI:** 10.3390/nu10050566

**Published:** 2018-05-04

**Authors:** Dimitrios Draganidis, Athanasios Z. Jamurtas, Theodoros Stampoulis, Vasiliki C. Laschou, Chariklia K. Deli, Kalliopi Georgakouli, Konstantinos Papanikolaou, Athanasios Chatzinikolaou, Maria Michalopoulou, Constantinos Papadopoulos, Panagiotis Tsimeas, Niki Chondrogianni, Yiannis Koutedakis, Leonidas G. Karagounis, Ioannis G. Fatouros

**Affiliations:** 1 School of Physical Education and Sport Science, University of Thessaly, Karies, 42100 Trikala, Greece; dimidraganidis@gmail.com (D.D.); ajamurt@pe.uth.gr (A.Z.J.); lavassia123@gmail.com (V.C.L.); delixar@pe.uth.gr (C.K.D.); kgeorgakouli@gmail.com (K.G.); guspapa93@gmail.com (K.P.); ptsimeas@gmail.com (P.T.); y.koutedakis@uth.gr (Y.K.); 2 School of Physical Education and Sports Science, Democritus University of Thrace, 69100 Komotini, Greece; stampoulistheodoros@gmail.com (T.S.); achatzin@phyed.duth.gr (A.C.); michal@phyed.duth.gr (M.M.); 3 First Department of Neurology, Aeginition Hospital, School of Medicine, National and Kapodistrian University, 11528 Athens, Greece; constantinospapadopoulos@yahoo.com; 4 Institute of Biology, Medicinal Chemistry and Biotechnology, National Hellenic Research Foundation, 116 35 Athens, Greece; nikichon@eie.gr; 5 Institute of Human Performance and Rehabilitation, Centre for Research and Technology—Thessaly (CERETETH), Karies, 42100 Trikala, Greece; 6 Faculty of Education Health and Wellbeing, University of Wolverhampton, Walsall 14287, West Midlands, UK; 7 Institute of Nutritional Science, Nestlé Research Centre, 1015 Lausanne, Switzerland; leonidas.karagounis@rdls.nestle.com; 8 Experimental Myology and Integrative Physiology Cluster, Plymouth Marjon University, Plymouth PL6 8BH, UK

**Keywords:** aging, chronic low-grade systemic inflammation, physical activity, nutrition, physical performance, chronic diseases

## Abstract

The development of chronic, low-grade systemic inflammation in the elderly (inflammaging) has been associated with increased incidence of chronic diseases, geriatric syndromes, and functional impairments. The aim of this study was to examine differences in habitual physical activity (PA), dietary intake patterns, and musculoskeletal performance among community-dwelling elderly men with low and elevated systemic inflammation. Nonsarcopenic older men free of chronic diseases were grouped as ‘low’ (LSI: *n* = 17; 68.2 ± 2.6 years; hs-CRP: <1 mg/L) or ‘elevated’ (ESI: *n* = 17; 68.7 ± 3.0 years; hs-CRP: >1 mg/L) systemic inflammation according to their serum levels of high-sensitivity CRP (hs-CRP). All participants were assessed for body composition via Dual Emission X-ray Absorptiometry (DEXA), physical performance using the Short Physical Performance Battery (SPPB) and handgrip strength, daily PA using accelerometry, and daily macro- and micronutrient intake. ESI was characterized by a 2-fold greater hs-CRP value than LSI (*p* < 0.01). The two groups were comparable in terms of body composition, but LSI displayed higher physical performance (*p* < 0.05), daily PA (step count/day and time at moderate-to-vigorous PA (MVPA) were greater by 30% and 42%, respectively, *p* < 0.05), and daily intake of the antioxidant vitamins A (6590.7 vs. 4701.8 IU/day, *p* < 0.05), C (120.0 vs. 77.3 mg/day, *p* < 0.05), and E (10.0 vs. 7.5 mg/day, *p* < 0.05) compared to ESI. Moreover, daily intake of vitamin A was inversely correlated with levels of hs-CRP (*r* = −0.39, *p* = 0.035). These results provide evidence that elderly men characterized by low levels of systemic inflammation are more physically active, spend more time in MVPA, and receive higher amounts of antioxidant vitamins compared to those with increased systemic inflammation.

## 1. Introduction

Chronic exposure to antigens as well as to chemical, physical, and nutritional stressors that the immune system has to cope with, in combination with the dramatic increase in life expectancy, result in the overstimulation of the immune system with advancing age and the development of a chronic and persistent pro-inflammatory state [1,2]. This age-associated, low-grade, chronic inflammatory status has been termed as “inflammaging” [1] and is clinically assessed by measuring systemic concentrations of cytokines and acute-phase proteins, including interleukin-6 (IL-6), tumor necrosis factor-α (TNF-α), and C-reactive protein (CRP) [3]. Inflammaging represents a significant risk factor for age-related frailty, morbidity, and mortality [2,4] as many chronic diseases and geriatric syndromes such as cardiovascular diseases, atherosclerosis, metabolic syndrome, type 2 diabetes mellitus, neurodegenerative diseases, cancer, and chronic obstructive pulmonary disease have been associated with chronic inflammation [5,6,7,8]. Moreover, increased levels of IL-6, TNF-α, and CRP in the elderly have been associated with lower muscle mass and physical performance [9,10,11] as well as with increased risk for sarcopenia and osteoporosis [12,13,14]. Thus, the concept of inflammaging appears to be a key determinant of successful aging and longevity and as such a valuable tool to counteract age-related pathologies [2].

To date, inflammaging is defined as a complex and multifactorial process whose origin cannot be simply attributed to a specific number of factors/mechanisms, as a complete understanding of the extent to which different tissues, organs, and biological systems contribute to its pathophysiology is lacking [3,15]. However, both physical activity (PA) and nutrition are considered powerful lifestyle factors that may, cooperatively or independently, influence both healthy aging and lifespan in humans [16,17]. Specifically, being physically active substantially reduces the risk of developing cardiovascular [16,17] and metabolic diseases [16,18], obesity [16,19], frailty [16,20,21], sarcopenia [22], osteoporosis [17,23], cognitive impairment [24], and mental health disorders [17,25] in a dose-response manner [26,27]. Numerous studies reported that higher volume of habitual PA is related to lower levels of IL-6, CRP, and TNF-α in older adults [28,29,30,31,32,33,34,35,36,37,38,39,40]. Most of these studies, though, are based on self-reported PA estimations [28,29,30,31,32,33,36,37,40] that may result in increased risk of recall bias [41] and therefore do not provide an objective determination of different intensity levels (i.e., light, moderate, vigorous, or very vigorous PA). However, to our knowledge, four studies have utilized accelerometry to provide an objective assessment of PA [34,35,38,39]. In two of them, an inverse relationship between PA and disease-related (chronic obstructive pulmonary disease and obesity) systemic inflammation was revealed in middle-aged adults [34,35]. Similarly, two other studies reported that time spent in MVPA is negatively associated with markers of systemic inflammation in the healthy elderly [38,39]. Although these data clearly suggest that habitual PA is inversely associated with mediators of systemic inflammation in older adults, a direct comparison of objectively assessed PA, sedentary time, and PA-related energy expenditure among the elderly with low and increased systemic inflammation is still lacking.

Ideally, this comparison would be more conclusive by the concurrent examination of habitual PA/inactivity and dietary intake levels, since both factors may impact systemic inflammation. In fact, available data suggest that the role of nutrition and dietary pattern is pivotal for immune function and low-grade systemic inflammation [42,43,44]. Both macronutrient and micronutrient intake may interfere with immune responses, triggering either a pro-inflammatory or an anti-inflammatory effect [45]. Excessive consumption of glucose and saturated fatty acids (SFA) (particularly long-chain SFA) are reported to activate pro-inflammatory markers in insulin-sensitive tissues [45,46] and may result in systemic inflammation [15], while high phospholipid consumption, especially that of polyunsaturated fatty acids (PUFA) and monounsaturated fatty acids (MUFA), elicit antiinflammatory properties and reduce the risk of chronic inflammation and its associated chronic diseases [47]. On the other hand, consumption of either plant- or dairy-based protein or amino acids may offer antiinflammatory effects by reducing levels of inflammatory mediators [45,48]. Furthermore, adequate intake of antioxidants and trace elements, particularly vitamins A, C, E, and selenium, also enhances immunity and elicits a protective effect against chronic inflammatory conditions [44]. However, to our knowledge, the literature lacks evidence regarding differences in dietary habits among older healthy adults with low and high systemic inflammation. 

Given the pivotal role of both PA and macronutrient/micronutrient intake in mediating immunity and chronic inflammatory responses, a direct comparison of them among older adults exhibiting low and elevated systemic inflammation may identify which parameters of these lifestyle factors function as discriminants of healthy aging and inflammaging. Therefore, the aim of the present study was to compare levels of objectively assessed habitual PA and dietary macronutrient/micronutrient intake, among otherwise healthy elderly men of low and increased systemic inflammation.

## 2. Materials and Methods

### 2.1. Experimental Design and Participants

A total of fifty community-dwelling elderly men aged 65–75 years were recruited from the surrounding area of Thessaly (Greece) through postings, newspaper, and media advertisements. All volunteers completed a health history questionnaire and were also examined by a physician. In order to be included in the study, volunteers had to initially meet all of the following inclusion/exclusion criteria: (a) nonsmokers; (b) independently living; (c) absence of chronic disease (i.e., cancer, metabolic, cardiovascular, neurological, pulmonary, or kidney disease); (d) absence of inflammatory disease (i.e., osteoarthritis, rheumatoid arthritis); (e) absence of type 2 diabetes, and (f) no recent or current use of antibiotics or other medication that could affect inflammatory status (i.e., corticosteroids). Subsequently, those who fulfilled these criteria underwent assessment of body height, body weight, body composition, handgrip strength, and physical performance (via the SPPB) testing to estimate their weight status and stage of sarcopenia according to the European Working Group on Sarcopenia in Older People (EWGSOP) [49]. Volunteers who were characterized as presarcopenic/sarcopenic were excluded from the study at this stage, since substantial loss of skeletal muscle mass is accompanied by significant performance decline [49], resulting in lower levels of habitual PA [50]. Volunteers who were classified as obese were also excluded since obesity is linked to metaflammation, an adipose-tissue-mediated chronic inflammatory state that differs in terms of pathophysiology from inflammaging [5,15]. Accordingly, thirty-four volunteers who fulfilled the eligibility criteria participated in the study. The determination of inflammatory status was based on two consecutive measurements of high-sensitivity CRP (hs-CRP) and participants were grouped as “low systemic inflammation” (LSI: hs-CRP < 1 mg/L) or “elevated systemic inflammation” (ESI: hs-CRP > 1 mg/L) according to a previous report [51]. Participants were then provided with accelerometers and food diaries to monitor their habitual PA and daily macronutrient/micronutrient intake, respectively, over a 7-day period. They were fully informed about the aim and the experimental procedures of the study, as well as about the benefits involved, before obtaining written consent. The Institutional Review Board of the University of Thessaly approved the study and all procedures were in accordance with the 1975 Declaration of Helsinki (as revised in 2000). 

### 2.2. Body Composition

Standing body mass and height were measured on a beam balance with stadiometer (Beam Balance-Stadiometer, SECA, Vogel & Halke, Hamburg, Germany) with participants wearing light clothing and no shoes as described previously [52]. Body composition [including fat mass, fat-free mass (FFM), percent of fat, lean body mass (LBM)] was assessed by dual emission X-ray absorptiometry (DXA, GE Healthcare, Lunar DPX NT, Diegem, Belgium) with participants in supine position as described before [53]. Appendicular lean mass (ALM) and skeletal muscle mass index (SMI) were calculated as the sum of muscle mass (kg) of the four limbs (based on DXA scan) and as ALM divided by height by meters squared (kg/m^2^), respectively [49], while sarcopenia status was determined according to the criteria established by EWGSOP [49]. 

### 2.3. Physical Activity

Physical activity was monitored by using the accelerometers ActiGraph, GT3X+ (ActiGraph, Pensacola, FL, USA) over a 7-day period. Accelerometers were attached to elastic, adjustable belts and did not provide any feedback to the participants. Participants were taught how to wear the belt around the waist with the monitor placed on the right hip and they were asked to wear it throughout the day, except for bathing or swimming and sleep, for seven consecutive days. To be included in the analysis, participants had to have ≥four days with ≥10 wear hours/day (i.e., four valid days) [54]. Nonwear time was calculated using the algorithms developed by Choi et al. [55] for vector magnitude (VM) data and defined as periods of 90 consecutive minutes of zero counts per minute (cpm), including intervals with nonzero cpm that lasted up to 2 min and were followed by 30 consecutive minutes of zero cpm. Daily activity and sedentary time were estimated according to VM data and expressed as steps/day and time in sedentary (<199 cpm), light (200–2689 cpm), moderate (2690–6166 cpm), vigorous (6167–9642 cpm), and moderate-to-vigorous (≥2690 cpm) PA [56]. The manufacturer software ActiLife 6 was utilized to initialize accelerometers and download data using 60-s epoch length. 

### 2.4. Dietary Assessment

Participants were taught by a registered dietitian how to estimate food servings and sizes of different food sources and how to complete food diaries. They were allowed to weigh out food servings, so that they could precisely report the amount of specific food portions, while they were also provided with colored photographs depicting different portion sizes that they could use to compare their food weights. Furthermore, complete instructions on how to describe portion sizes based on household measures or other standard units were also administered to our participants. Participants recorded their daily dietary intake for seven consecutive days, describing, in as much detail as possible all portions of food and drinks/water. For commercially available products, the name of the manufacturer, fat content (i.e., 1%. 2%, etc.), and other related information had to be noted. The Science Fit Diet 200 A (Science Technologies, Athens, Greece) dietary software was utilized to analyze diet recalls and data regarding total energy (kJ), protein (g/kg/day & g/day), leucine (g/day), branched chain amino acids (BCAA, g/day), carbohydrates (g/day), fat (g/day), vitamin A (IU/day), vitamin C (mg/day), vitamin E (mg/day), selenium (μg/day), polyunsaturated fatty acids (PUFA), and monounsaturated fatty acids (MUFA). 

### 2.5. Systemic Inflammation

Blood samples were collected early in the morning between 07:00 and 09:00 am, after an overnight fasting. Participants were asked to avoid alcohol and abstain from intense physical activity for ≥48 h before blood sampling. Blood was drawn from an antecubital arm vein via a 10-gauge disposable needle equipped with a Vacutainer tube holder (Becton Dickinson) with participants seated. To separate serum, blood samples were allowed to clot at room temperature and then centrifuged (15,000× *g*, 15 min, 4 °C). The supernatant was dispensed in multiple aliquots (into Eppendorf tubes) and stored at −80 °C for later analysis of hs-CRP. Serum hs-CRP was quantitatively measured in duplicate using the C-Reactive Protein (Latex) High Sensitivity assay (CRP LX High Sensitive, Cobas^®^) on a Cobas Integra^®^ 400 plus analyzer (Roche) with a detectable limit of 0.01 mg/dL and an inter-assay coefficient of one standard deviation (1 SD).

### 2.6. Statistical Analyses

All data are presented as means ± SD. The normality of data was examined using the Shapiro–Wilk test (*n* = 17/group). Because our data sets in most of our variables differed significantly from normal distribution, we rejected the hypothesis of normality and applied nonparametric tests. To test differences in body composition, daily PA-related parameters, and dietary macronutrient/micronutrient intake among the two groups (LSI vs. HSI) a Kruskal–Wallis test was applied. Pearson’s correlation analysis was used to examine the relation of dietary antioxidant vitamins intake, number of steps, and time in MVPA per day with serum levels of hs-CRP. Correlation coefficients of *r* < 0.2, 0.2 < *r* < 0.7 and *r* > 0.7 were defined as small, moderate, and high, respectively. Effect sizes (ES) and confidence intervals (CI) were also calculated for all dependent variables using the Hedge’s g method corrected for bias. ES was interpreted as none, small, medium-sized, and large for values 0.00–0.19, 0.20–0.49, 0.50–0.79, and ≥0.8, respectively. The level of statistical significance was set at *p* < 0.05. Statistical analyses were performed using the SPSS 20.0 software (IBM SPSS Statistics). The G * Power program (G * Power 3.0.10) was utilized to perform power analysis. With our sample size of 17/group we obtained a statistical power greater than 0.80 at an α error of 0.05.

## 3. Results

Participants’ characteristics are presented in Table 1. Participants were healthy and had no pathological levels of hs-CRP. The two groups, though, differed significantly in respect to hs-CRP values (ESI: 2.1 ± 0.8 vs. LSI: 0.7 ± 0.2 mg/dL, *p* = 0.00), with ESI displaying a 2-fold elevation in serum hs-CRP compared to LSI. Averaged BMI values in LSI and ESI were 27.3 ± 3.1 kg/m^2^ and 27.9 ± 2.5 kg/m^2^, respectively, which classifies them as nonobese according to the criteria established by the World Health Organization (WHO) [57]. Moreover, all participants were characterized as nonsarcopenic, since they exhibited SMI > 7.26 kg/m^2^, handgrip strength > 30 kg, and physical performance score in SPPB > 8. No differences were detected in respect to BMI, fat mass, percent of fat, FFM, LBM, ALM, SMI, and handgrip strength among groups. However, significant differences were observed in physical performance, with LSI achieving a higher SPPB score compared to ESI (LSI: 11.9 ± 0.2 vs. ESI: 11.2 ± 1.0; *χ*^2^ = 6.436, *p* = 0.016; ES = 0.90; 95% CI = −1.63, −0.17).

**Figure 1:**
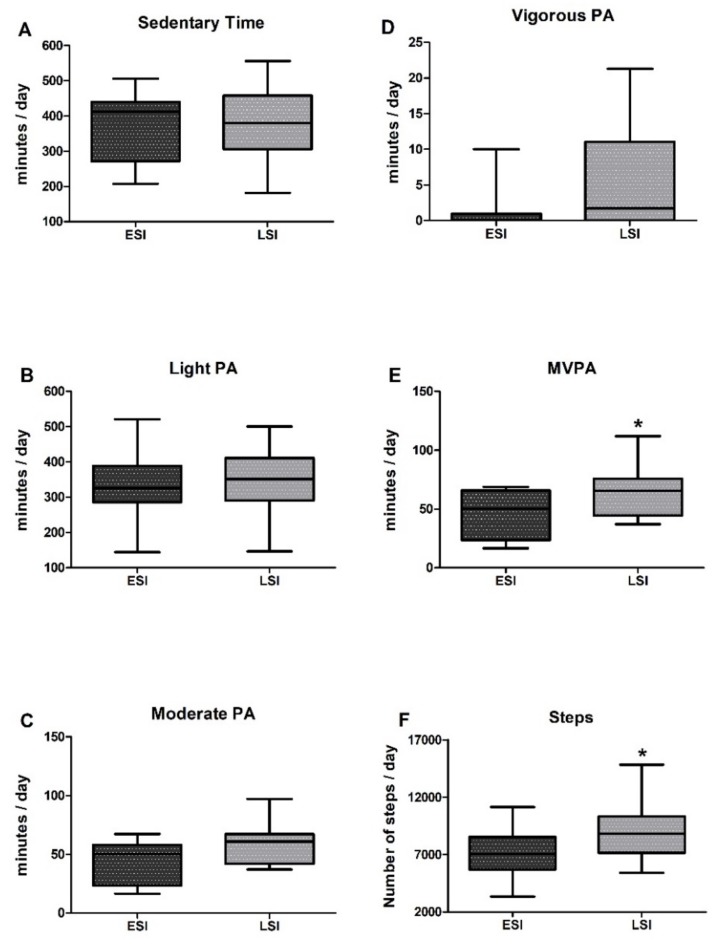
(**A**) Sedentary time, (**B**) time spent in light, (**C**) moderate, (**D**) vigorous, (**E**) moderate-to-vigorous (MVPA) PA, and (**F**) total step count throughout the day, in low (LSI) and elevated (ESI) systemic inflammation groups. Values are presented as mean ± SD. * denotes significant difference between groups at *p* < 0.05.

Results comparing sedentary time and PA among groups are shown in Figure 1. The two groups were comparable in sedentary time throughout the day (LSI: 378.2 ± 98.7 vs. ESI: 370.5 ± 95.9 min/day; *χ*^2^ = 0.008, *p* = 0.927) and in the time they spent in light PA/day (LSI: 342.9 ± 93.1 vs. ESI: 331.7 ± 98.2 min/day; *χ*^2^ = 0.357, *p* = 0.550), while a trend for significantly more time spent in moderate PA/day by the LSI group was also observed (LSI: 59.5 ± 16.7 vs. ESI: 44.1 ± 18.2 min/day; *χ*^2^ = 3.637, *p* = 0.057). Interpretation of the level of moderate PA by group means examined in relation to the PA guidelines adopted by the WHO revealed that both groups met the recommendation for at least 150 min of moderate-intensity PA throughout the week.

**Figure 2:**
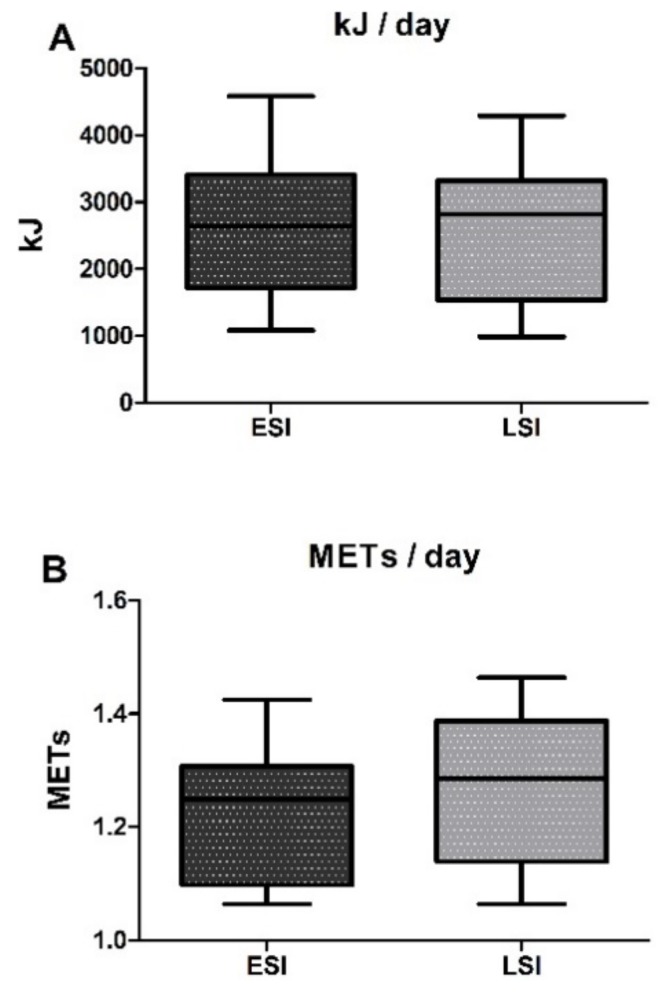
Daily PA-related energy expenditure expressed as (**A**) kJ and (**B**) METs in low (LSI) and elevated (ESI) systemic inflammation groups. Values are presented as mean ± SD.

By performing an individual examination in both groups, we found that all participants in LSI and approximately 86% of participants in ESI met this criterion. Significant differences between LSI and ESI were observed in MVPA and daily step count, with LSI spending more time in MVPA throughout the day (LSI: 65.2 ± 21.5 vs. ESI: 45.9 ± 19.8 min/day; *χ*^2^ = 3.997, *p* = 0.044; ES = 0.91; 95% CI = −1.68, −0.13) and performing more steps (LSI: 9000.1 ± 2496 vs. ESI: 6968.3 ± 2075 steps/day; *χ*^2^ = 4.087, *p* = 0.043; ES = 0.86; 95% CI = −1.63, −0.08) than ESI, by 42% and 30%, respectively. The average step count/day for LSI was 9000.1 steps, which is close to the upper recommended limit for older adults (7100–10,000 steps/day) [58] while the ESI did not meet these recommendations, performing 6968.3 steps/day. Almost 86% of participants in the LSI group performed >7100 steps daily while slightly more than half (53%) of participants in the ESI group did so. A longitudinal analysis combining both groups revealed a trend for an inverse correlation between hs-CRP level and daily step count (*r* = −0.37, *p* = 0.055). Time in vigorous PA/day did not differ among groups (LSI: 5.3 ± 6.9 vs. ESI: 1.0 ± 2.6 min/day; *χ*^2^ = 2.315, *p* = 0.128), probably because of a high interindividual variability. Moreover, the two groups demonstrated similar PA-related energy expenditure throughout the day, as no differences observed in terms of kJ/day (LSI: 2554.3 ± 1033.5 vs. ESI: 2654.3 ± 1041.8 kJ/day, *p* = 0.798) and METs/day (LSI: 1.28 ± 0.1 vs. ESI: 1.23 ± 0.1 METs/day, *p* = 0.203) (Figure 2).

**Figure 3:**
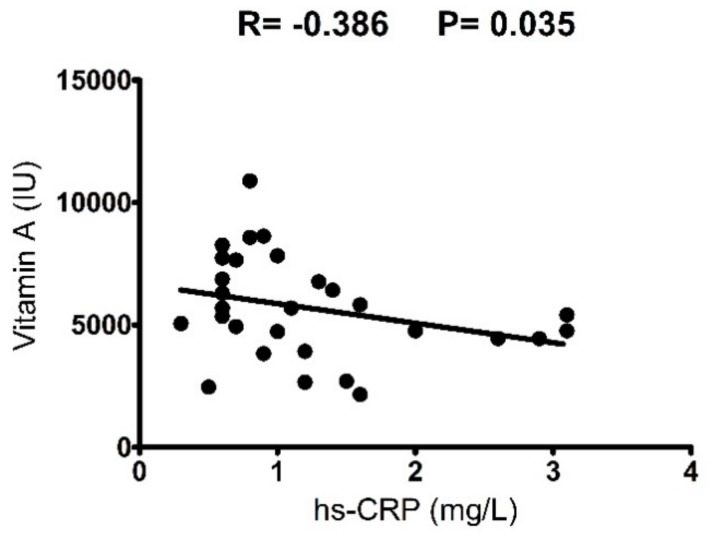
The relationship between serum hs-CRP level and daily dietary intake of Vitamin A.

LSI and ESI demonstrated similar total energy and macronutrient intake throughout the day (Table 2). The two groups had a daily energy intake of 6949.6–6794.8 kJ, constituted by 15–16% protein, 38% carbohydrate, and 42% fat. The mean protein intake in both groups was 0.8 g/kg body weight/day, which represents the recommended daily allowance (RDA) that meets 97.5% of the population [59]. However, approximately 46% of participants in both groups had a daily protein intake of 0.5–0.7 g/kg body weight/day. Separate analysis in leucine and BCAA intake revealed that both LSI and ESI received 0.6 g of leucine/kg body weight/day and 0.13–0.14 g of BCAAs/kg body weight/day, which meets the current recommendations for amino acid intake in adults [59]. The two groups, though, differed significantly in respect to daily antioxidant vitamin intake, with the LSI group receiving higher amounts of vitamin A (LSI: 6590.7 ± 2219 vs. ESI: 4701.8 ± 1552.6 IU/day; *χ*^2^ = 5.616, *p* = 0.018; ES = 0.95; 95% CI = 1.72, 0.18), vitamin C (LSI: 120.0 ± 55.5 vs. ESI: 77.3 ± 39.1 mg/day; *χ*^2^ = 5.421, *p* = 0.020; ES = 0.87; 95% CI = 1.63, 0.11), and vitamin E (LSI: 10.0 ± 2.9 vs. ESI: 7.5 ± 3.0 mg/day; *χ*^2^ = 4.496, *p* = 0.034; ES = 0.75; 95% CI = 1.50, 0.01) than ESI, by 37%, 59%, and 33%, respectively. Moreover, by performing a longitudinal analysis of both groups we observed that daily vitamin A intake was inversely correlated with levels of hs-CRP (*r* = −0.39, *p* = 0.035) (Figure 3). On the contrary, daily intake of selenium (LSI: 93.2 ± 29.8 vs. ESI: 96.1 ± 29.7 μg/day, *p* = 0.793), PUFA (LSI: 10.1 ± 2.4 vs. ESI: 8.9 ± 2.6 g/day, *p* = 0.215), and MUFA (LSI: 43.7 ± 10.8 vs. ESI: 37.9 ± 10.9 g/day, *p* = 0.168) was comparable in the two groups.

## 4. Discussion

The present study is the first, to our knowledge, to compare the levels of habitual PA, sedentary time, and dietary intake between healthy elderly men with low and elevated low-grade systemic inflammation (inflammaging). Our findings suggest that older adults characterized by low levels of systemic inflammation perform more steps and spent more time in MVPA throughout the day and they receive higher amounts of dietary antioxidant vitamins (i.e., vitamins A, C, and E) on a daily basis compared to their counterparts with elevated systemic inflammation.

Participants were categorized as having either “low” or “elevated” low-grade systemic inflammation according to their serum levels of hs-CRP. This acute-phase protein is considered a valid and informative marker of inflammaging [60] and has been previously used as a single marker to identify levels of systemic inflammation in older adults [51]. The term inflammaging, first introduced by Franceschi and his colleagues [1], refers to the development of a chronic, low-grade inflammation phenotype with advancing age. However, the presence of obesity, either in young or older individuals, results in elevated systemic inflammation, which has been defined as metaflammation (metabolic inflammation) and is primarily mediated by the adipose tissue [5]. Although the underpinning mechanisms of inflammaging and metaflammation may be different, these two chronic inflammatory conditions may overlap [15]. Therefore, in an attempt to focus on inflammaging in this study, we included only nonobese elderly men (according to WHO criteria). Moreover, LSI and ESI groups were very homogeneous in terms of body composition, since they did not differ in body weight, fat mass, percent of fat, FFM, and LBM. All participants were also nonsarcopenic according to the criteria established by the EWGSOP [49], since the existence of sarcopenia could act as a covariate in our investigation, interfering with their ability to habitually perform PA [50]. 

Previous cross-sectional studies have investigated the association between habitual PA and inflammatory biomarkers in middle-aged and older adults [28,29,30,31,33,34,35,36,37,38,39,40]. However, only two utilized accelerometry to quantify not only the quantity but also the quality (intensity) of habitual PA in the otherwise healthy elderly with physiological and elevated chronic, low-grade systemic inflammation [38,39]. This study attempted to extend the current literature by providing insights concerning the differences in PA and dietary intake profile among elderly men with low and elevated low-grade systemic inflammation. The use of accelerometry to objectively assess the quantity and intensity of habitual PA is a strength of our study, as most of the previously cited studies [28,29,30,31,33,36,37,40] are based on questionnaires, self-reports, or interviews. The use of accelerometers over a 7-day period to assess PA and sedentary time has been reported to be a valid and reproducible methodological approach in the elderly [61].

Although sedentary time and time spent in light- and moderate-intensity activities throughout the day were similar between LSI and ESI, we noted that overall the LSI group performed more steps and spent more time in MVPA on a daily basis. This suggests that not only the volume of habitual PA but also the intensity in which daily physical activities are performed may interfere with the development of chronic, low-grade systemic inflammation in older individuals. Our findings further build on previous reports that higher volume of habitual PA is associated with lower levels of pro-inflammatory mediators in healthy elderly individuals [29,33,36] and COPD patients [34]. Moreover, this inverse association between PA and inflammation is suggested to be dose-dependent, so that the more physically active an individual is, the lower the chronic inflammatory milieu [29,31,40]. Although only a trend (*r* = −0.37, *p* = 0.055) for an inverse correlation between hs-CRP level and daily step number was observed in our study, possibly because of an interindividual variability in daily step counts of our participants (we used accelerometers whereas questionnaires were utilized by others), these findings collectively suggest that habitual PA may be associated with inflammaging in an inverse, dose-response pattern. Furthermore, it has been recently reported that the impact of PA on chronic low-grade inflammation is not only dose-dependent but also intensity-dependent, as moderate-to-vigorous activities induce greater improvements in the inflammatory profile of older adults while light- or moderate-intensity physical activities are accompanied by no changes in inflammatory mediators [62]. Indeed, Wahlin-Larsson et al. [39] found that in recreationally active elderly women, the time spent in MVPA is inversely associated with serum levels of CRP, a finding also reported in younger individuals [63]. The mechanism/s through which PA reduces or prevents low-grade systemic inflammation in the elderly remains to be elucidated. Observational, cross-sectional studies are not designed to identify the mechanisms that underline the effects of systematic PA on chronic inflammation and as such, more intervention studies are needed [41,62]. Based on the fact that inflammaging is tightly regulated by the balance between pro- and anti-inflammatory mediators [64], a possible mechanism could be that PA, and especially MVPA, suppresses the production of pro-inflammatory cytokines and molecules that trigger the inflammatory milieu, and enhances the production of anti-inflammatory mediators [41,62,65]. Moreover, the process of inflammaging may be further affected by the age-associated increase in the production of reactive oxygen and nitrogen species (RONS) that lead to redox balance disturbances and subsequent activation of the redox-sensitive NF-κB signaling pathway that stimulates the expression of numerous pro-inflammatory mediators such as TNF-α, IL-6, IL-1β, and CRP [48,66]. As such, a vicious cycle of RONS and pro-inflammatory molecule production is propagated, driving a chronic systemic pro-inflammatory phenotype [48,67]. Regular participation in moderate-to-vigorous intensity exercise has been shown to attenuate both basal and exercise-induced levels of oxidative damage, enhance the antioxidant capacity, and improve the DNA repair machinery in healthy, elderly individuals [68,69]. Thus, it can be proposed that systematic MVPA may prevent the development of inflammaging by lowering the production of RONS and levels of oxidative damage in the elderly.

LSI and ESI also differed significantly in terms of physical performance. More specifically, LSI exhibited higher performance in the SPPB test compared to ESI and this observation is in line with previous findings reporting that older adults with elevated systemic inflammation demonstrate lower physical performance [70,71]. Although the underlying mechanism leading from chronic inflammation to functional decline has not been clarified yet, it has been reported that systemic inflammation may impact physical performance by decreasing skeletal muscle mass [14,48]. However, in this study, the two groups demonstrated similar LBM, ALM, and SMI, indicating that the observed difference in physical performance was not muscle-mass-dependent. A previous report, though, by Wahlin-Larsson and colleagues [39] provided evidence that increased systemic inflammation influences muscle regeneration by decreasing the proliferation rate of myoblasts. In addition, increased inflammation and cytokine production may also reduce the quiescent satellite cells pool and attenuate their differentiation capacity [14]. Therefore, it can be assumed that elevated systemic inflammation may contribute to physical performance deterioration by attenuating the regeneration potential of the aged skeletal muscle.

We also utilized 7-day recalls to perform a thorough screening of the dietary intake in the LSI and ESI groups, focusing on macronutrients and micronutrients that have been shown to elicit either a pro- or an anti-inflammatory effect, and could be therefore characterized as ‘key modifiers’ in the process of inflammaging. LSI and ESI demonstrated similar energy and macronutrient intake, consuming 6794.8–6949.6 kJ/day composed of 15–16% protein, 38% carbohydrates, and 42% fat. Our group recently conducted a literature review suggesting that protein intake, especially that of whey protein and soy or isoflavone-enriched soy protein, may indirectly offer antioxidative and anti-inflammatory benefits beyond its ability to stimulate skeletal muscle protein synthesis [48]. Also, Zhou et al. [72] performed a meta-analysis on the effects of whey protein supplementation on levels of CRP, concluding that increased whey protein intake may induce favorable effects on individuals with elevated baseline CRP levels. However, in this study, we noted that daily protein intake was similar between LSI and ESI, with both groups receiving on average ~0.8 g/kg BM/day, which is in line with WHO RDA for protein [59]. BCAA and leucine intake were also compared among groups to provide a qualitative determination of daily protein intake. Although leucine is classified as a BCAA, we decided to present it separately because its role may differ from that of the other BCAAs, especially in the elderly where a higher amount of leucine should be consumed through diet to efficiently stimulate muscle protein synthesis and preserve muscle loss [73,74]. In our present work, we observed that LSI and ESI had a similar daily intake of BCAAs and leucine, meeting the recommendations for amino acid intake in adults [59]. Daily carbohydrate intake was also similar among groups (154–156 g/day), indicating that it does not play a prominent role in the development of inflammaging. Previous reports have noted that only increased consumption of high glycemic index carbohydrates may be associated with increased levels of inflammation [75]. Unfortunately, the determination of glycemic index and glycemic load in our participants’ daily diets was not feasible.

Similarly, no differences were observed in total fat consumption among groups, with LSI and ESI receiving 79 and 74 g/day, respectively, which corresponds in both groups to 42% of daily energy intake. Although previous reports have indicated that increased fat consumption is associated with elevated systemic markers of inflammation [75,76], this was not the case here. High fat diets, and primarily SFA, have been reported to induce substantial alterations in the gut microbial flora (i.e., increases gut mucosa permeability, epithelial brier disruption) that result in enhanced translocation of lipolysaccharide (LPS) in the circulation, thus promoting the development of low-grade systemic inflammation [76,77]. However, it should be highlighted here that not all SFA demonstrate equal properties and consumption of specific SFA (i.e., C14:0, C15:0, C17:0, CLA, and trans-palmitoleic) has been associated with positive effects on cardiovascular health [78]. On the other hand, increased intake of MUFA and/or PUFA has been proposed to counteract the pro-inflammatory cascade by reducing the translocation of LPS in the circulation [76] and suppressing the eicosanoid and PAF inflammatory pathways [47]. Indeed, many studies have revealed an inverse association between higher intake of dietary PUFA and/or MUFA and levels of pro-inflammatory mediators such as hs-CRP and IL-6 [75]. In this study, although no statistically meaningful differences were observed in dietary MUFA and PUFA intake between groups, LSI displayed a higher intake of MUFA and PUFA, by 15% and 13.5%, respectively, compared to ESI.

Interestingly, we noted significant differences between LSI and ESI in terms of antioxidant vitamin intake. More specifically, daily dietary intake of vitamins A, C, and E in LSI was higher by 37%, 59%, and 33%, respectively, as compared to ESI. These vitamins play a major role in immune function, so that adequate intake enhances innate, cell-mediated, and humoral antibody immunity while deficiency promotes the opposite effects [44,79]. With aging, the production of reactive oxygen and nitrogen species and that of pro-inflammatory cytokines rises significantly, propagating a vicious cycle of oxidative stress and inflammation that promotes a chronic low-grade inflammatory state [48,67]. Vitamin A has been shown to promote a T-helper type 2 immune response by reducing the expression of pro-inflammatory cytokines (i.e., interferon-γ, TNF-α and IL-12) and adipocytokines (i.e., leptin) [44,79] while it may also inhibit the activation of the redox-sensitive nuclear factor-kappa B (NF-κB) [44,79], a principal mediator of the bidirectional interaction between oxidative stress and inflammation [48]. Moreover, the pivotal role of vitamin A in chronic inflammation is further supported by the fact that a deficit in vitamin A intake is associated with a pronounced pro-inflammatory state and inability to cope with pathogens, as well as with reduced phagocytic capacity of macrophages [44]. Vitamin C also reduces the production of pro-inflammatory cytokines through inhibition of the transcription factor NF-κB [44]. The anti-inflammatory effect of this micronutrient is further supported by a previous investigation where vitamin C intake was inversely associated with levels of CRP and tissue plasminogen activator (t-PA) antigen in elderly men [80]. Furthermore, vitamin C acts as a potent antioxidant, protecting cells from ROS-mediated oxidative damage, while it may also boost the synthesis of other antioxidants such as vitamin E [44]. Likewise, vitamin E is able to confer protection against oxidative stress by increasing the concentration of endogenous antioxidant enzymes, such as SOD, CAT, and GPX, and it also prevents oxidative damage in the cell membrane [44,81]. Evidence based on human studies indicates that vitamin E supplementation in older adults improves immune function [44] and is associated with a lower concentration of pro-inflammatory mediators [82]. Collectively, these data corroborate the higher antioxidant vitamin intake observed in LSI in the present study, indicating that vitamins A, C, and E may contribute to the control of low-grade systemic inflammation in the elderly. By contrast, no differences were observed in selenium intake between LSI and ESI, although selenium is also considered a micronutrient that may efficiently influence both innate and acquired immune function and may enhance the antioxidative defense system [44].

## 5. Conclusions

We found that elderly men with low levels of systemic inflammation are characterized by higher quality and quantity of habitual PA and ingested higher amounts of antioxidant vitamins A, C, and E through normal diet when compared to those with increased systemic inflammation. To the best of our knowledge, this is the first study to directly compare elderly men of low and increased low-grade systemic inflammation in respect to habitual PA and dietary profile. PA and antioxidant vitamin intake appear to be discriminant factors of inflammaging and healthy aging. Future research should further explore the cause and effect as well as the dose-response relationship between PA and/or antioxidant vitamins and inflammaging.

## Figures and Tables

**Table 1:** Participants’ characteristics.

Parameter	LSI (*n* = 17)	ESI (*n* = 17)
Age (years)	68.2 ± 2.6	68.7 ± 3.0
Body Height (m)	1.71 ± 0.07	1.73 ± 0.04
Body Weight (kg)	82.3 ± 8.5	85.2 ± 7.5
BMI (kg/m^2^)	27.3 ± 3.1	27.9 ± 2.5
Fat Mass (kg)	24.1 ± 7.0	26.3 ± 4.1
Fat (%)	29.5 ± 6.6	31.8 ± 2.1
Fat-Free Mass (kg)	56.3 ± 4.6	58.4 ± 5.2
Lean Body Mass (kg)	53.3 ± 4.5	55.3 ± 5.1
ALM (kg)	23.2 ± 2.4	24.4 ± 2.1
SMI (kg/m^2^)	8.12 ± 0.7	8.13 ± 0.6
Grip Strength (kg)	34.3 ± 5.5	36.7 ± 6.6
SPPB (score)	11.9 ± 0.2	11.2 ± 1.0 ^1^
Sarcopenia Status	Non-Sarcopenic	Non-Sarcopenic
hs-CRP (mg/L)	0.7 ± 0.2	2.1 ± 0.8 ^2^

Data are presented as mean ± SD. ALM: Appendicular Lean Mass; SMI: Skeletal Muscle Mass Index; SPPB: Short Physical Performance Battery; hs-CRP: High-Sensitivity CRP. ^1^ significant difference between groups, *p* < 0.05, ^2^ significant difference between groups, *p* < 0.01.

**Table 2:** Dietary macronutrient and micronutrient intake in LSI and ESI groups.

Parameter	LSI (*n* = 17)	ESI (*n* = 17)	*p* Value	*χ*^2^
Total Energy (kJ/day)	6952.9 ± 1241.8	6797.8 ± 1136.8	0.771	0.085
Protein				
g/day	63.8 ± 20.3	66.9 ± 14.6	0.183	1.770
g/kg BM/day	0.8 ± 0.3	0.8 ± 0.2	0.817	0.054
% of total calories	15 ± 2.7	16 ± 3.0		
Leucine (g/day)	4.89 ± 1.7	5.13 ± 1.2	0.430	0.624
BCAAs (g/day)	11.38 ± 3.6	11.53 ± 2.4	0.533	0.389
Carbohydrates				
g/day	156.2 ± 37.6	154.9 ± 52.7	0.901	0.016
% of total calories	37.7 ± 6.9	37.5 ± 8.4		
Fat				
g/day	79.3 ± 12.5	73.7 ± 17.0	0.318	0.996
% of total calories	42.0 ± 4.0	41.7 ± 7.1		
PUFA (g/day)	10.1 ± 2.4	8.9 ± 2.6	0.275	1.191
MUFA (g/day)	43.7 ± 10.8	37.9 ± 10.9	0.359	0.840
Vitamin A (IU/day)	6590.7 ± 2219.6	4701.8 ± 1552.6 ^1^	0.018	5.616
Vitamin C (mg/day)	120.0 ± 55.5	77.3 ± 39.1 ^1^	0.020	5.421
Vitamin E (mg/day)	10.0 ± 2.9	7.5 ± 3.0 ^1^	0.034	4.496
Selenium (μg/day)	93.2 ± 29.8	96.1 ± 29.7	0.589	0.292

Data are presented as mean ± SD. BM: Body mass; BCAA: Branched chain amino acids; PUFA: Polyunsaturated fatty acids; MUFA: Monounsaturated fatty acids. ^1^ Significant difference between groups.
